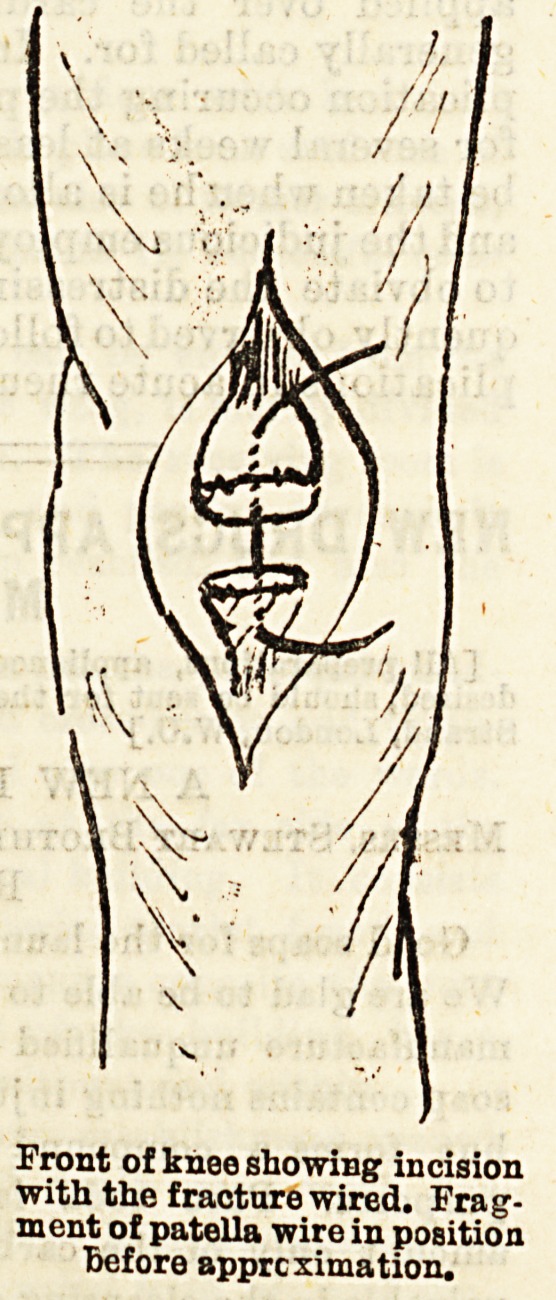# Treatment of Fractured Patella

**Published:** 1893-04-08

**Authors:** 


### BRISTOL GENERAL HOSPITAL.

### Treatment of Fractured Patella.

Simple fractures of the patella may be divided, for purposes of treatment, into two classes: (1) Where the fibrous expansion from the quadriceps tendon which covers the anterior surface of the bone is intact, or at least not completely torn through, and thus separation of the fragments to any extent is prevented. This condition occurs very rarely when the bone is broken by muscular action, but is the rule in fracture due to a direct blow. (2) Where this fibrous expansion is completely torn, allowing of, and necessitating, an appreciable separation of the fragments. This (by far the commonest kind of fracture) is the result of a sudden contraction of the powerful quadriceps in an attempt to save a fall with the knee semiflexed, the patella in this position of the joint resting only by the central portion of its articular surface against the femur, and so being snapped across.

In the 1st class the treatment is simple and effectual, there being no separation of the fragments to be overcome. Bony union readily takes place with any form of apparatus that keeps the knee fixed for four or five weeks.

In the 2nd class the treatment adopted here is of two kinds—non-operative and operative.

(1) *Non-operative Treatment.*—The usual method is to approximate the fragments (after subsidence of the initial swelling by simple rest on a straight splint) by means of two broad pads of lint placed transversely across the limb just above and below the patella (an assistant holding the fragments together), and fixing these on by several strips of soap plaster passing over the upper pad from above downwards and backwards, and over the lower pad in the reverse direction—trying to avoid any tilting of the fragments by pressure in front of their edges—and bandaging the leg and thigh to a long straight back splint with a foot-piece, reaching as far as the gluteal fold.

At other times a Croft's plaster of paris splint is applied instead of the straight splint, enclosing the limb entirely, except the patella. Another method that has been tried is as follows: Two rectangular pieces of poro-plastic are cut of sufficient breadth to half surround the thigh and leg respectively, and a deep U-shaped notch is made in each, the bottom of the U

Poroplastic pieces, separately and in position:

being fitted to the circumference of the fragments. These pieces are moulded upon the thigh and leg (after being softened by warming before a fire), the limbs of the U overlapping one another and accurately enclosing the patella between them. The pieces are then bandaged firmly on, and the whole limb placed on a straight splint.

(2) *Operative Treatment.*—The only procedure adopted is that of wiring the fragments together by Lister's open method. The time that is allowed to elapse after the accident before the operation is done varies from within the first twenty-four or thirty-six hours to a week or ten days, when the fluid part of the blood and the inflammatory effusion have had time to subside. It is also undertaken as a secondary operation for a useless or painful knee after failure of simpler treatment.

The details of the operation as practised here are as follows: In the morning of the operation the knee and adjoining half of the thigh and leg are thoroughly washed and surrounded by a piece of lint soaked in carbolic lotion (1 in 20), which is covered with some waterproof material—such as a "pink jaconet"—and bandaged on. At the operation all instruments are put to soak in carbolic lotion (1 in 20)—preferably for half an hour before use—a mackintosh is spread under the limb and covered with a towel wrung out of the same lotion, and the area of operation is isolated in the same way by carbolised towels. The knee is finally well sponged with the lotion, and the surgeons and assistants' hands (and especially nails) are carefully cleansed and carbolised. A free vertical incision is made over the patella, the fragments freed from the overlying tissues, and any clot or fluid removed from the joint. The fractured surfaces of both fragments will be found to be tilted forward and covered with blood clot, and a thick fringe of the torn aponeurotic expansion and periosteum turned in over their edges. This fringe is cut away flush with the edge, and the fractured surface scraped with a Volkmann's spoon or raspatory till two flat bony surfaces are obtained which can be brought accurately together. The fragments are then bored by an ordinary bradawl in a slanting direction from the anterior surfaces, the point being brought out on the fractured surfaces as near to the cartilaginous edge as possible, and accurately opposite one another. Pure flexible silver wire ("medium-size patella wire") is used, and it is generally fairly easy to make each end of the wire follow the point of the bradawl as this is withdrawn from the fractured surface. Before approximating the fragments, a dependent opening is made over a pair of sinus-forceps pushed down into the joint behind the external lateral ligaments, and a medium-sized drainage tube is made to project slightly into the joint. The joint is then sponged with carbolic lotion (1 in 40), and the fragments drawn together, the wire being twisted three or four times, and the end hammered down over the upper fragment. The skin wound is closely sutured, and a small drainage tube inserted at the lower angle. This is covered with a thin strip of "protective," and the whole knee covered with carbolic gauze, well wrung out of carbolic lotion (1 in 40), (at least eight layers are necessary for the first dressing), a thick layer of corrosive sublimate wood wool (Hartmann's), and an "outside" carbolic gauze dressing. The leg is placed on a long straight splint with a foot-piece.

Front of knee showing incision with the fracture wired. Fragment of patella wire in position before approximation.

The dressings are changed the next day (the first oozing being very copious), when, if all is well, the drainage tubes are removed and the same dressings re-applied for five or six days. If the posterior drainage tube is left in more than twenty-four hours, the second dressing must be sooner than this. The wounds generally heal soundly under the third or fourth dressing—that is, a fortnight after the operation. Passive movements are now begun daily—the splint being left off—and in three weeks the patient can get up with crutches and begin active movements. Active and passive manipulation must be persevered in resolutely.

In comparing the results of the two methods of treatment, the great advantage of the latter is in the strength of the knee. Non-operative treatment gives, as a rule, a very good immediate result, with often freer flexibility of the joint at first, but it rarely stands the test of time, the ligamentous union gradually stretching as the limb is used. In the operative treatment adopted here no misadventure has ever happened, the confinement to bed and the length of the treatment is minimised, and the stiffness beyond flexion to a right angle, which is often left for some time, generally wears off gradually, if slowly, and leaves the patient with a knee as strong as it was before.

## Figures and Tables

**Figure f1:**
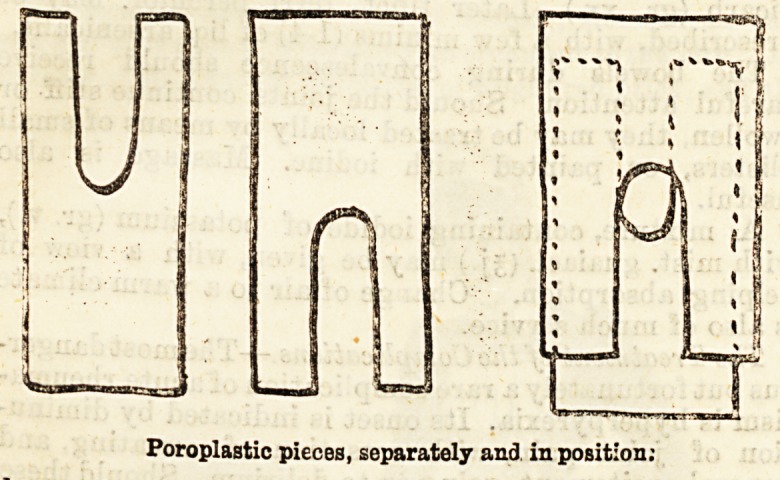


**Figure f2:**